# Impact of an Okinawa/Nordic based diet on endocrinological and periodontal conditions in individuals with type 2 diabetes. A randomized case–control study

**DOI:** 10.1186/s12903-023-03272-9

**Published:** 2023-08-09

**Authors:** G. Rutger Persson, Cecilia Widén, Björn Wohlfart, Klas Sjöberg, Stig Steen, Michael D. Coleman, Helene Holmer

**Affiliations:** 1 Departments of Periodontics, and Oral Medicine, School of Dentistry, University of Washington, HSB Box 357444, Seattle, WA 98195 USA; 2 Faculty of Health, Kristianstad University, SE-29188 Kristianstad, Sweden; 3 Department of Cardiothoracic Surgery, Clinical Sciences, Lund University, SE-22100 Lund, Sweden; 4 Department of Gastroenterology, Lund University, Lund, Sweden; 5 Department of Cardiothoracic Surgery, Clinical Sciences Lund, Lund University, SE-22100 Lund, Sweden; 6 School of Life and Health Sciences, Aston University, Birmingham, UK; 7 Kristianstad Central Hospital, SE-29185 Kristianstad, Sweden

**Keywords:** Diet, Diabetes mellitus, HbA1c, Gingivitis, Periodontitis, Cytokine

## Abstract

**Objectives:**

To assess if the results following intake of a diet using an Okinawan-based Nordic diet (OBND) over one month differs in endocrinological, periodontal clinical outcome, and serum cytokine levels compared to a standard hospital care diet in individuals with diabetes type 2 (T2D) (control group).

**Background:**

Scientific evidence suggests that the use of diet for individuals with T2D may be beneficial.

**Methods:**

Participating individuals with T2D were randomly assigned to a test (OBND) (*n* = 14), or control group (*n* = 16). Anthropometric data, blood glucose levels, HbA1c levels, lipids, serum inflammation markers (CRP, and a routine panel of 24 cytokines), blood pressure, gingival bleeding on probing (BOP), probing pocket depths (PPD), and clinical attachment levels (CAL) were studied.

**Results:**

Statistical analyses of baseline study data failed to demonstrate study group differences. The mean weight reduction was greater in the OBND group (4.1 kg) versus the control group (1.3 kg) (*p* < 0.01). The reduction in BMI was 1.4 kg/m2 in OBND (*p* < 0.001) and 0.5 kg/m2 in the control group, respectively (*p* < 0.01). Diastolic and systolic blood pressure reductions were greater in the OBND group than in the control group (*p* < 0.01). Periodontal study parameters (BOP % scores) and (PPD values) decreased (*p *< 0.001) overall with no between group differences. The OBND resulted in reduction of serum levels of IFNγ, Eotaxin IL-9, IP10,IL17a, MCP-1,m and PDFBB compared to the control diet.

**Conclusions:**

A strict T2D- diet provides an association between reduction in serum HbA1c and BOP scores. Serum levels decreases in IFNγ, Eotaxin IL-9, IP-10, IL17a. MCP-1, and PDFBB were only found in the test group.

**Supplementary Information:**

The online version contains supplementary material available at 10.1186/s12903-023-03272-9.

## Introduction

Diet, genetic factors, and a healthy lifestyle, may explain why Okinawa islanders are renowned for their longevity [1]. Data suggest that an Okinawan-based Nordic diet (OBND) consistent with Scandinavian preferences provide similar clinical benefits as a designated diet from Okinawa [2, 3]. While rich in fiber and proteins, the specific OBND is characterized by low calorie, and carbohydrate levels. The source of proteins is mainly derived from beans/lenses/peas, oily fish, and poultry. The OBND is also rich in vitamins and minerals but with low refined carbohydrates levels.

Recent data suggest that individuals with T2D consuming a diet based on the OBND concept may reduce body weight, improve blood lipids, and glucose levels [2]. Furthermore, data suggest that in a healthy non-diabetic study group consuming a breakfast consistent with an OBND and compared to a conventional breakfast, reduced postprandial rise in glucose and insulin levels [3]. Other studies have also demonstrated that the OBND after a period of 12 weeks consumption can reduce sweet cravings, and increasing satiety, with improved metabolic status, reduced serum levels of inflammatory markers, and with positive changes in gastrointestinal hormone secretions [3–7]. In one study, the reduction of bleeding on probing (BOP), using a diet consistent with OBND was consistent with what can be anticipated from a one-time performed professional non-surgical standard of care periodontal treatment of gingival inflammation [8].

Many studies demonstrate that a diagnosis of gingivitis and/or periodontitis is common in individuals with T2D. According to the Swedish National Diabetes Care guidelines (2015) medical professionals should refer patients for dental treatment in cases of persistent gingivitis or periodontitis [9]. The literature on the association between periodontitis and T2D is extensive showing that worsening of glycemic control can be caused by chronic inflammation [10–18]. Furthermore, data suggest that individuals with T2D and periodontitis commonly have elevated serum LPS-induced levels of IL-6, IL-8, IL-10 [19]. Data also suggest that a palaeolithic diet may improve oral health while reducing inflammation in healthy individuals and in the absence of performing routine oral hygiene procedures [20].

The aim of the present study was to assess if (I) short-term tangible benefits with OBND in terms of medical and dental health outcomes over a short study period, and if (II) the study outcomes differed between individuals with T2D using OBND compared to a standard hospital care diet suitable for individuals with T2D (control group diet). Primary outcome measures were: changes in serum HbA1c levels, and the severity of gingival inflammation as assessed by evidence of bleeding on probing (BOP). Secondary outcomes were changes in serum levels of cytokines.

## Materials and methods

### Study population and selection criteria

Consistent with Swedish law and the Helsinki Declaration of 1964 with all amendments, the Swedish Ethical Review Authority Regional Ethical Committee in Lund, Sweden (Institutional Review Board case no. 2017/540) approved the study. The study was performed 2017. Informed consent was obtained from those who were recruited from the Health Care Center (HCC) connected to the Endocrinology Clinic at Kristianstad Central Hospital, Sweden. Clinicians involved in the care of potential study participants were neither involved in the recruitment, nor did they perform study procedures. Study individuals were randomly assigned to the test (OBND), or to the standard of care diet (control group). The assignment to groups were made by the HCC using a computer assigned data program.

The study inclusion criteria were: (I) study individuals age ≥ 18 years, and (II) having a confirmed diagnosis of T2D. The exclusion criteria were: (I) current need for antibiotic treatment, or a history of treatment with antibiotics during the preceding month, (II) a systolic blood pressure > 180 mm Hg, (III) having visited a dental clinic within 3 months prior to study start, (IV) being a current smoker, snuff user, (V) currently pregnant, (VI) suffering from rheumatoid arthritis, (VII) being diagnosed with lactose- or gluten intolerance, or (VIII) having any other severe co-morbidity. A total of 199 potential study individuals with T2D were recruited from medical records with a follow up period of ≥ 5 years and with stable serum Hb1Ac. A total of 30 individuals gave written consent to participate.

### Study design

The study is a short-term randomized pilot case–control study including individuals with a diagnosis of T2D and using insulin. The randomization was performed by a study group coordinator otherwise not associated with the study and assigning subjects to groups depending on when the participants could come to the first examination. The test group received OBND. The control group received a standard of care diabetic designed diet. Prior to study start, and at end of study after 4 weeks, blood samples were collected and analyzed at the Kristianstad Hospital Medical Laboratory. Pertinent data regarding diabetic conditions and complications were collected from current patient records after the clinical examination had been performed. This was done by an experienced endocrinologist. Thereafter, all participants were examined by an experienced periodontist (GRP). Neither before the first, nor at the final examination were the medical and dental clinical examiners informed about study group assignments and without access to medical dental previous data. During 2018, all clinical examinations were performed at the Central Hospital in Kristianstad, Sweden. Following the medical examinations, the dental examinations were performed at the clinical dental office at the Central Hospital in Kristianstad. All examinations were performed at the same time of the day as the first and final serum samplings were performed, Dental examinations were performed at the Oral Surgery clinic at Kristianstad Central Hospital. No treatment of endocrinological or dental conditions were performed during the study. A licensed periodontist who has performed many clinical trials including calibration procedures (GRP) performed the clinical dental examinations. The examiners could not be blinded to whether study participants had started, or completed the study. This was due to the restrictions on how the meal portions were distributed. The blood analyses were, however, performed blinded as to study sequence.

The OBND food was produced by Igelösa Life Science AB, Lund, Sweden. The standard hospital care diet (control) was produced by Blekedamm AB, Kristianstad, Sweden. The diet was delivered individually by a truck delivery service. During the study period, all participants received daily packages of prepared food given study assignments using specially designed cooling bags. These packages were distributed such that neither delivery personnel or recipients could identify provided diet. The bags with potential left-over food were returned through the delivery service to control for compliance. In not one single case was it possible to identify non-compliance.

### Medical analyses

All medical examinations and blood sample collections were performed at the time of enrollment and at study endpoints but before clinical examinations. Blood samples were analyzed at the Department of Clinical Chemistry, and at the Department of Internal Medicine at the Central Hospital in Kristianstad, Sweden. In addition, the individuals were examined in respect to general state of health.

### Periodontal examination

The dental examinations, and oral sample collections were performed at the time of enrollment and at study endpoint examinations. The following recordings were performed: probing of pocket depths with a manual mm graded periodontal probe (PPD)s were assessed at four sites per tooth, clinical attachment levels (CAL; were also assessed at four sites per tooth), and bleeding on probing (BOP; assessed at four sites per tooth). PPD and CAL measurements were made with periodontal probes (PCR-12, Hu-Friedy Corp, Chicago, IL, USA). The clinical assessments of probing depths, and clinical attachment levels were performed by a periodontist (GRP) calibrated from a several preceding studies and with clinic experiences over 40 years. The extent of bleeding on probing was measured approximately 30 s after probing of pocket depths. The extent of BOP was assessed, and a diagnosis of gingivitis was declared if ≥ 10% and in accordance with the guidelines provided by the AAP (American Academy of Periodontology), and the EFP (European Federation of Periodontology) [17–21].

### Diet

An example of a one-week diet based on the OBND diet principle is provided (Tables 1 and 2). The nutritional facts for the OBND diet and for the hospital standard of care diet are presented (Table 3).

**Table 1:** Examples of the Okinawan-based Nordic diet

**Raw vegetables**						
For breakfast 100 g in a salad, for lunch 150 g and for dinner 150 g						
**Breakfast**						
Porridge with berries and milk or sour milk with berries and muesli, open face sandwich with cheese, turkey ham and 100 g of mixed vegetables						
**Lunch**						
Day 1	Day 2	Day 3	Day 4	Day 5	Day 6	Day 7
Carrot and coconut- milk soup, with red lentiles, shrimps and whole intact grains	Salmon with saffron gravy, ratatouille and whole intact grains	Broiled chicken leg, brown gravy, potatoes, cauliflower, broccoli, carrots and cabbage	Oven- baked codfish, cold mustard gravy, broccoli, cauliflower and whole intact grains	French vegetable soup with basil pesto and whole intact grains	Pea soup with rooster sausage and onion, cold mustard	Swedish sausage Stroganoff, black whole grain rice, green peas
**Afternoon snack**						
Apples & walnuts	Pears & fruit truffles	Orange & almonds	Apples & fruit truffles	Pears & walnuts	Orange & almonds	Pears & Plum-cake
**Dinner**						
Oven- baked sausage potato mash, broccoli, salad	Meat stew, mixed grilled vegetables, whole intact grains	Whole grain pasta with chicken, ground meat gravy	Potato patties with feta cheese and sundried tomatoes grilled vegetables, tomato cream gravy	Cabbage pudding, brown gravy, lingonberry, grilled vegetables	Vienna sausage, turnip mash, sauerkraut, whole intact grains with green peas	Oatmeal nuggets, mixed grilled vegetables, tamari soy sauce

**Table 2:** Energy composition, energy percent (E%), of OBND®, standard of care diet and recommendations for diabetes diets based on the Nordic Nutrition Recommendations (NNR 2012)

**Nutritional variable**	**OBND®**	**Standard of care**	**Recommended NNR 2012**
Energy (kJ)	5984	8424	
Energy (kcal)	1432	2015	
Fat (E%)	31	41	25–40
-saturated fat (E%)	9	15	< 10
-mono-unsaturated fat (E%)	12	14	10–20
-poly-unsaturated fat (E%)	8	7	5–10
Carbohydrate (E%)	41	38	45–60
Fiber (g/MJ)	5	3	3
Protein (E%)	22	18	10–20

**Table 3:** Energy composition, energy percent (E%), of OBND®, standard of care diet and recommendations for diabetes diets based on the Nordic Nutrition Recommendations (NNR 2012)

**Nutritional variable**	**OBND®**	**Standard of care**	**Recommended NNR 2012**
Energy (kJ)	5984	8424	
Energy (kcal)	1432	2015	
Fat (E%)	31	41	25–40
-saturated fat (E%)	9	15	< 10
-mono-unsaturated fat (E%)	12	14	10–20
-poly-unsaturated fat (E%)	8	7	5–10
Carbohydrate (E%)	41	38	45–60
Fiber (g/MJ)	5	3	3
Protein (E%)	22	18	10–20

### Serum cytokine analyses

A commercially available cytokine panel (Bio-Rad, Sundbyberg, Sweden) was used to assess cytokine levels in serum including; Basic FGF (basic fibroblast growth factor), Eotaxin, GCSF (granulocyte colony‐stimulating factor), IFNγ (interferon gamma), Interleukin (IL): IL1β (interleukin 1 beta), IL1ra (receptor antagonist), IL4, IL5, IL6, IL7, IL8, IL9, IL10, IL12p70 (active heterodimer), IL13, IL17A, IP10 (interferon‐inducible protein‐10), MCP1 (monocyte chemo‐attractant protein‐1), MIP1α (macrophage inflammatory protein 1 alpha), MIP1β (macrophage inflammatory protein 1 beta), PDGF‐BB (platelet‐derived growth factor subunit B), TNFα (tumor necrosis factor alpha), and VEGF (vascular endothelial growth factor) were assayed with the Luminex MagPix multi analyte technology (Luminex, Austin, TX, USA). The manufacturer’s instructions were followed. The researcher who performed the cytokine analysis was unaware of clinical data, and followed the instructions as provided by the manufacturer and laboratory guidelines. A high level of agreement between measurements were identified following replicated readings for a subset of samples demonstrating (intra‐class correlation (ICC) varying between 0.95 and 1.0 (*p* < 0.001).

### Statistical analyses

The statistical package SPSS 25 was used for all the analyses. All analyses were performed with masked (coded) study group assignment and revealed after completion of data analyses. The statistical methods used in the present study included Chi-square tests, odds-ratio calculations, independent samples t-tests (equal variance not assumed), paired samples t-tests (equal variance not assumed), and bivariate correlation analyses (Pearsons’ correlation). Oneway ANOVA (Post-hoc Bonferroni) tests were also included as appropriate. The significance level was defined at *p* < 0.05. Based on pilot data from similar studies which were available to the investigators, changes in bleeding scores were considered sufficient to declare study group differences at *p *< 0.05 if 15 individuals were enrolled in each study group. Binary regression analysis was performed to search for explanatory variables. The focus of the present study was to assess changes regarding the extent of gingival bleeding, serum HbA1c (% change) as well as differences (/% change) in serum levels of cytokines studied between groups.

## Results

All consenting, and enrolled study individuals completed the study. A consort checklist is available as supplemental information.

### Study characteristics

The test group included 14 study individuals with T2D (7 females). The control group included16 study individuals (10 females). The average time since the diagnosis of T2D was 12.5 years (SD 9.7 years, range 2 to 38 years with 24 individuals with T2D ≥ 6 years). The average age of the study population was 68.6 years ± (SD 6.4 years) with no statistical difference between study groups. The female study participants were between age 58 and 71 and menopausal. In the present study, none of the participants was lost to follow-up. No complaints regarding foods supplied were reported. Data analyses using independent t-tests (equal variance not assumed) and baseline data failed to demonstrate differences by study group assignments for all clinical medical and dental variables. Baseline values for medical and dental variables are presented (Table 4).

### Anthropometric data

Data analyses based on paired t-tests identified statistically significant weight reduction during the study period respectively in both groups. The weight reduction was, however, significantly greater in the test group (4.2 kg) than in the standard of care group (15 kg) (*p* < 0.01). The reduction in BMI was 1.4 kg/m^2^ in test group (*p* < 0.001) and 0.5 kg/m^2^ in the control group (*p* < 0.01) (significantly greater in the OBND group). The difference in waist circumference was also greater in the OBND group (*p* < 0.01 (Table 4).

**Table 4:** Mean levels and standard deviation (S.D.) of key variables for individuals Okinawan based Nordic Diet (OBND) or Standard of Care Diet (*n* = 16). Paired samples T-test

**Medical data**	**OBND (*n* = 14)**				**Sign**	**Standard of care (*n* = 16)**				**Sign**
	**Baseline** **(week 0)**		**At study endpoint** **(week 4)**			**Baseline** **(week 0)**		**At study endpoint** **(week 4)**		
	**Mean**	**S.D**	**Mean**	**S.D**		**Mean**	**S.D**	**Mean**	**S.D**	
*Anthropometric data*										
Weight (kg)	96.3	18.9	92.2	18.4	*p* < 0.001	86.9	18.1	85.6	18.0	*p* < 0.01
Waist circumference (cm)	110.2	14.1	106.6	14.9	*p* < 0.001	105.7	15.8	104.7	15.0	NS
BMI (kg/m^2^)	33.3	6.8	31.9	6.7	*p* < 0.001	30.6	6.6	30.1	6.4	*p* < 0.01
*Blood pressure & pulse*										
Syst.blood pressure (mm Hg)	139.8	19.0	129.0	16.7	*p* < 0.05	145.8	22.0	143.8	22.5	NS
Diast. blood pressure (mm Hg)	80.4	13.5	73.1	8.5	*p* < 0.01	82.7	10.8	82.7	10.3	NS
Pulse (beats per min)	73.9	13.0	68.9	11.7	NS	76.6	12.7	74.2	10.5	NS
*Blood sugar & lipids*										
HbA1c (mmol/mol)	56.7	16.1	50.6	11.2	*p* < 0.001	57.4	9.9	54.6	9.2	*p* < 0.001
Cholesterol (mmol/L)	4.5	1.3	3.4	1.1	*p* < 0.001	4.1	1.0	3.7	0.9	*p* < 0.01
LDL-cholesterol (mmol/L)	2.7	1.2	1.9	1.0	*p* < 0.001	2.5	0.9	2.2	0.9	*p* < 0.01
HDL-cholesterol (mmol/L)	1.3	0.3	1.2	0.2	NS	1.2	0.4	1.2	0.4	NS
Triglycerides (mmol/L)	2.3	1.7	1.6	0.9	*p* < 0.05	2.1	1.3	1.8	0.9	NS
*Inflammation*										
CRP (mg/L)	3.1	2.7	2.7	2.8	NS	2.7	3.1	2.8	3.7	NS
Thrombocytes (10^9^/L)	244.1	56.9	232.2	53.2	NS	243.5	65.4	238.8	62.4	NS
Leucocytes (10^9^/L)	6.7	1.8	6.4	1.7	NS	6.9	1.2	6.7	1.6	NS
Creatinine	82.1	25.1	78.9	20.8	NS	73.7	14.8	73.8	13.3	NS
Hemoglobin	133.3	14.3	131.3	10.9	NS	140.4	12.1	137.3	11.5	NS
*Periodontal health*										
Bleeding on probing (%)	17.7	13.5	11.6	15.0	*p* < 0.05	29.0	26.2	18.4	23.4	*p* < 0.001
Pocket depth 1 mm (%)	37.1	17.4	64.6	22.5	*p* < 0.001	39.2	18.1	72.0	26.5	*p* < 0.001
Pocket depth 2 mm (%)	54.4	14.4	34.4	21.8	*p* < 0.01	55.7	14.0	26.4	23.9	*p* < 0.001
Pocket depth 3 mm (%)	6.8	6.1	0.6	1.0	*p* < 0.001	4.4	4.9	1.2	2.9	*p* < 0.001
Pocket depth 4 mm (%)	1.1	1.7	0.2	0.6	*p* < 0.05	0.4	0.9	0.3	1.0	NS
Pocket depth 5 mm (%)	0.3	0.5	0.2	0.4	NS	0.1	0.4	0.1	0.5	NS
Pocket depth > 5 mm (%)	0.3	1.3	0.0	0.0	NS	0.2	0.6	0.0	0.0	NS

### Medical and dental conditions at baseline and study endpoint (within study group analyses) (Table 4)

Analyses by independent t-tests (equal variance not assumed) failed to demonstrate baseline differences between study groups for all clinical and dental variables studied. Mean values, standard deviations for key medical and periodontal study variables at baseline and at study endpoint are presented and identifying within group changes over time as analyzed by paired t- tests (equal variance not assumed). The presented results suggest similar changes for paired t-tests identified similar and statistically significant reductions in serum HbA1c levels, cholesterol, and low-density lipoproteins (LDL) levels (*p* < 0.001). High density lipoprotein levels (HDL) were not significantly changed in either study group. Other blood samples taken to evaluate general health of the participants (data not shown) showed normal values both at baseline and at study endpoint (sodium and potassium, creatinine, hemoglobin, leukocyte, thrombocyte, and serum CRP levels) in both study groups.

Dental data are also presented (Table 4). On average, the individuals in the OBND and control groups had similar numbers of remaining teeth (OBND mean: 24.7; S.D. ± 2.9) (control group mean: 23.9; S.D. ± 3.7) (*p* = 0.524). At baseline, bleeding on probing in the OBND and control groups ≥ 20% was identified in 32.9%, and 50.0%, respectively, which changed at the study endpoint to 14.3% and 25.0%, respectively (*p* < 0.001). Statistical analyses failed to demonstrate differences in clinical PDs or CALs measurements between baseline and study endpoint for both study groups. According to the new classification system periodontitis baseline data identified periodontitis in the OBND, and control groups at stage III, in 28.5% (4 individuals), and 18.8% (3 individuals), respectively.

### Medical and dental conditions at baseline and study endpoint (between study group analyses)

The changes in medical conditions differed between study groups for the following variables: weight, waist circumference, diastolic blood pressure, and LDL levels (Table 5). The reported changes (reductions) were greater in the OBND group (Table). Most statistical comparisons failed, however, to report differences between study groups (values not reported). Thus, similar changes between baseline and study endpoint for the % BOP, and reduction in PPD depth (mm changes), as well as serum HbA1c level were found between study groups (NS). Data analysis by Pearson Chi square also failed to demonstrate group differences by the overall periodontal diagnosis based on the new classification system (*p* = 0.22).

**Table 5:** Changes between baseline and study endpoint for the OBND and control groups for clinical study variables and selected serum cytokines(independent t-test equal variance not assumed)

**Medical and dental variables**	**OBND group**		**Control group**		**S.E. diff**	**95% CI**		**Sign**
	**Mean**	**S.D**	**Mean**	**S.D**				
Diast.blood pressure (mm Hg)	-7.8	8.7	0.4	9.2	3.3	-15.0	-1.6	0.02
Weight (kg)	-4.2	2.7	-1.54	1.8	0.8	-4.4	-1.0	0.001
Waist circumf. (cm)	-3.3	2.8	-0.8	2.,5	0.97	-4.5	-0.5	0.01
BMI	-4.2	2.7	-1.5	1.8	-2.7	-4.1	-0.9	0.01
Cholesterol (mmol/L)	-22.6	11.7	-9.2	10.1	4.0	-21.6	-5.3	0.05
LDL-cholesterol (mmol/L)	-31.2	17.8	-12.1	16.4	6.3	32.0	-6.3	0.01
Eotaxin	-13.2	25.6	12.6	20.5	-25.8	-43.5	8.6	0.01
IFN-γ	-31.0	35.8	11.7	39.2	14.3	-71.7	-13.0,	0.05
IL 17a	-21.1	22.9	5.1	16.2	-26.2	-40.9	-11.5	0.01
IP 9	-20.5	25.0	-1.8	12.7	-18.8	-33.6	7.2	0.05
IP 10	-24.6	29.1	13.3	21.2	-37.9	-72.2	.3.6	0.05
MCP-1	-18.6	39.4	10.2	22.2	-28.9	-53.8	11.9	0.05
PDGFBB	-13.6	21.2	3.3	13.1	-16.9	-30–5	-3.3	0.001

### Serum cytokine changes

**Fig. 1:**
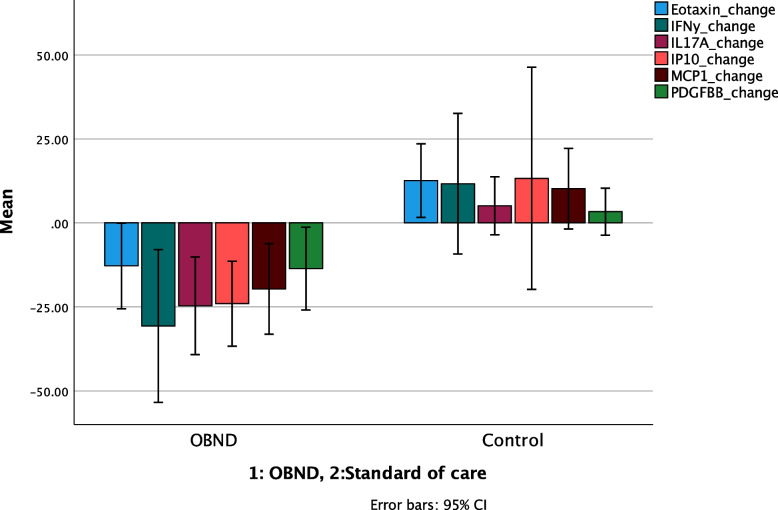
Mean values and error bars 95% (CI.) for changes in selected serum cytokines with differences in changes between before and at the post study examinations between study groups

Between group analyses for changes in serum cytokine levels identified different patterns of change by study groups Thus, independent t-tests (equal variance not assumed) identified significant decreases for the following serum cytokines in the OBND group compared to the control group for; serum Eotaxin (*p* < 0.001), Il-17A (*p* < 0.001), IFN-γ (*p* < 0.05), Il-9 (*p* < 0.05), IP10(< 0.05), MCP1 (*p* < 0.05), and PDFBB (*p* < 0.05 (Table 5, and Fig. 1).

**Fig. 2:**
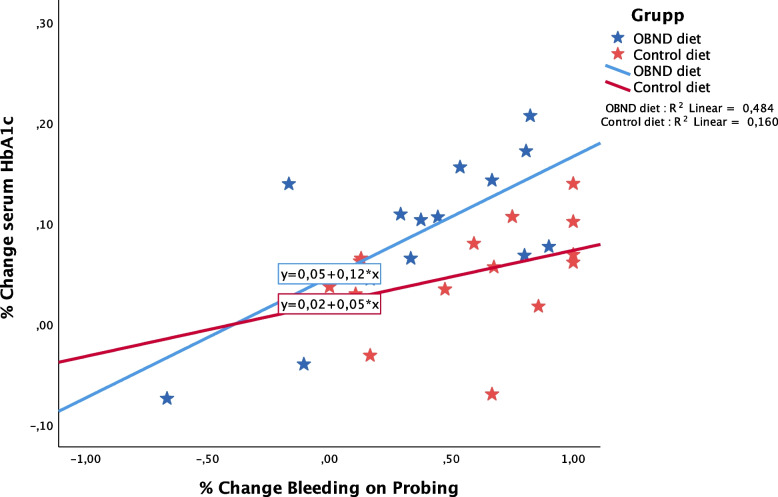
Correlation between % changes in serum HbA1c levels and % changes in bleeding on probing by study groups

3.5 Changes in cytokine levels and changes (%) in BOP, and/or serum HbA1c for the OBND group. Analyses by Pearsons´ correlation identified a significant correlation between changes between BOP, and/or serum HbA1c level changes (*p *< 0.01) ( Fig. 2). Statistical analyses failed to demonstrate significant associations between changes in these two variables in relation to serum cytokine levels. No significant associations between changes in BOP% and serum HbA1c were found in the control group. Furthermore, statistical analyses failed to demonstrate associations between changes in either %BOP, or serum HbA1c to cytokine changes in both groups.

Binary regression analysis was identified that only the change (reduction) of serum LDL levels differed significantly between study groups to the advantage of the OBND diet (*p* < 0.001). If a ≥ 5% reduction of serum HbA1c was considered as a successful outcome (otherwise not) and independent of diet such data analysis failed to demonstrate differences in study variable changes.

## Discussion

The Okinawan-based Nordic diet is consistent with the Nordic official nutrition standards as defined in 2012. The food was well accepted by all the participants. All participants complied with the study requirements (based on assessments of returns and questionnaires). None of the study individuals was lost to follow-up. A strength of the present study was that all daily packages with prepared meals were delivered by dedicated carriers (unaware of what they provided). To ensure compliance and to encourage study completion, those who delivered the food were in regular contact with study participants. To further improve the compliance partners/spouses were also provided the diet and at no costs to them. These individuals were not part of the study. The present study was conducted before the onset of the COVID19 pandemic. The study identified, however, that home deliveries of individually prepared food with daily rations would over a longer time periods be too expensive and with several logistic restrictions. It is possible that the knowledge obtained during the pandemic and the common deliveries of both groceries and prepared food might have given us information that would have facilitated the present study.

All study participants had a long track record of supportive diabetic care and had as part of the inclusion requirements stable diabetic conditions. Clinically relevant, and similar improvements were obtained in both study groups. The overall 64% reduction in the extent of gingival bleeding during a one-month study period with no professional oral hygiene instruction or treatment is a clinical important observation. The clinical improvements cannot simply be explained as a Hawthorne or bias effect. The reduction in % BOP as identified was also linked to improvements in serum HbA1c levels as well as for reduced levels of some pro-inflammatory cytokines. The present study also demonstrated that the control diet designed to meet standard of care diet to any individual with a diagnosis of T2D had similar clinical outcome impact as the OBND diet. the improvement in serum HbA1c values were correlated to the improvement in the extent of gingival inflammation. One explanation to the clinical improvements in gingival conditions may be explained by the general sugar restriction by the two diets. Most likely, early bacterial colonization primarily by streptococci species may have had favorable effects on the extent and severity of gingival inflammation [20].

The present study also showed that body weight, waist circumference, BMI scores, blood lipids i.e., cholesterol, LDL and TG were improved in both study groups but significantly more so in the OBND group, which had a lower calorie content. The individuals in the OBND group had, on average, a higher BMI score at baseline. The potential to reduce body weight might have been an advantage for weight reduction. The reduced levels of pro-inflammatory cytokines may be associated with the relative loss of weight especially the OBND group. The statistically significant reductions in the OBND group for Eotaxin Il-17A IFN-γ Il-9 IP10MCP1 and PDFBB and for clinical parameters suggest that the OBND appeared to have relevant advantages over the control diet.

The impact of diet as a factor on cardiovascular conditions has been identified among Japanese individuals and people using a Mediterranean dietary [22–24]. The present study demonstrated that although serum HbA1c levels were lower in the OBND group at baseline, this group obtained a further reduction in serum HbA1c levels greater than in the standard of care study control group. During the study period, the test diet in the OBND group reduced diastolic blood pressure whereas the standard of care of diet was less effective in reducing systolic blood pressure.

Both the OBND, and the hospital standard of care diet had a proven positive impact on the clinical progress of the clinical conditions. The OBND diet had, in several aspects, better effects on endocrinological parameters and serum cytokine levels than the control hospital diet. To validate the present findings, future studies should monitor the effects of diet over longer time periods. A true control group of individuals with no clinical evidence of T2D should also be included. Indeed, clinical changes regarding both medical and dental parameters may, in fact, require longer periods than 30 days to confirm that clinically relevant changes were obtained. The greater the proportional reduction in serum HbA1c levels, as well as the greater the reduction in the proportion of bleeding on probing, suggests a strong relationship between serum HbA1c and severity of gingival inflammation as defined by clinical bleeding on probing in the present study. This was more pronounced in the OBND test group. Notwithstanding, data suggest that using a ketogenic diet improves both glycemic and lipid profiles in individuals with T2D [25].

The lack of association between changes in serum level for cytokines studied and changes in the extent of BOP is consistent with finding from other studies for the control group [26–28]. The impact of the dedicated diet (OBND) provided evidence of improvements in gingival conditions as observed clinically consistent with changes in serum levels of pro-inflammatory cytokine. The reduction in % BOP in the present study was also associated with improvements in serum HbA1c levels. Other studies have shown associations between reduction of gingival inflammation and corresponding effects on serum cytokine levels (.i.e. IL-17A, Eotaxin, MCP-1, and IFN gamma) [29–33]. The present study failed to identify correlation between changes in these serum parameters and changes in BOP%. This may be related to the short study period.

The primary shortcomings of the present study were the limited 30 days study period, and that only 30 study participants were included in the study. Given the fact that serum HBA1c levels routinely are monitored as a method to the get an average estimate of blood sugar levels of longer periods than 30 days we did not expect to show immediate effects on blood sugar levels. Nevertheless, the short-term diet (OBND) for individuals with T2D had an impact on potential diabetes risks such as body weight. Body weight was reduced over this short period. Additional studies over perhaps six months may be required to observe serum Hb A1c level changes. Longer periods may be needed to study sustainable effects of the OBND diet.

The present report similar results as two other studies [34, 35]. It is consistent with current guidelines advising people with prediabetic and diabetic conditions to lose weight [36, 37]. To obtain results reducing periodontal probing depths, and a possible gain of clinical attachment levels it would be necessary to extend the study period used in the present study to maybe one year. A control study population of study individuals who did not have a diagnosis of diabetes but placed on the same OBND diet would also be of valuable inclusion in a new study protocol.

The concept of receiving “ready to eat” food for a continuous period of 30 days was unfortunately not acceptable to many potential study individuals. The present study was also restricted by the IRB regarding study length and absence of conventional therapy for diagnosed treatment needs beyond the study period of 30 days. It is possible that in the context of the COVID 19 experiences the concept of delivered ready to eat meals would be more appreciated.

The Okinawan-based Nordic diet is high in nutrition and fiber, but low in calories. Notwithstanding, the diet provides good satiety for a prolonged time. The Okinawan-based Nordic diet promotes the use of a wide variety of ingredients. The main characteristic is high nutritional value and fiber content which provides long-lasting satiety with a minimum calory amount. It is based on whole intact grains – including oat, barley, rye, wheat, and millet, plenty of vegetables of all colors, root fruits, beans, and legumes, fish, and some poultry, vegetable oils, fruits, berries, nuts, and seeds. The diet is also restrictive in sugar, salt, red meat, processed meat, but also fatty dairy products.

The diet complies with the Nordic Nutrition Recommendations as well as many international official dietary recommendations. In comparison with the Nordic nutrition recommendations, the macronutrient profile of the Okinawan based Nordic diet (OBND) used in the present study has a slightly higher protein and fiber contents above the Nordic recommendations. The fat content in the OBND is, however, in accordance with the current recommendations. In contrast, the OBND carbohydrate content is slightly below those recommendations.

From a metabolic point of view, the Okinawa diet resembles in the Mediterranean diet with regards to fibers, vegetable oils, fruits, berries. The dietary fibers content is beneficial for the digestion. From a cardiovascular perspective, the unsaturated fat, and the antioxidant content should contribute to lowering levels of inflammation. Furthermore, sugar, salt, processed meat, and saturated fats restrictions of the OBND diet are in accordance with current WHO recommendations, and other established health authorities. The low sugar content and low total caloric diet is also consistent with current efforts to reduce blood sugar levels, and control of insulin needs among individuals with T2D.

## Conclusions

Intake of a dedicated diet is effective on the management of diabetic and periodontal conditions. The study identified that both study groups reduced body weight, lowered serum HbA1c levels and reduced gingival inflammation. Data suggested that the improvements were more pronounced in the OBND group. The gingivitis reduction was correlated to serum HbA1c reduction. The reduction of pro-inflammatory serum cytokine levels was greater in the OBND over the control diet in managing T2D and inflammation of the gingival tissues.

### Supplementary Information

**Additional file 1.**

## Data Availability

Whereas additional material is under analysis the investigators are limited in what they might provide public access to. Future publication may be affected. Select information can be provided given rational why access to the databases is necessary. Information will be granted once requested from the p.i. (email: rper@uw.edu).
